# Draft Genome Sequences of Thelohania contejeani and Cucumispora dikerogammari, Pathogenic Microsporidia of Freshwater Crustaceans

**DOI:** 10.1128/MRA.01346-20

**Published:** 2021-01-14

**Authors:** Alexandre Cormier, Rémi Wattier, Isabelle Giraud, Maria Teixeira, Frédéric Grandjean, Thierry Rigaud, Richard Cordaux

**Affiliations:** a Laboratoire Ecologie et Biologie des Interactions, Université de Poitiers, UMR CNRS 7267, Poitiers, France; b Laboratoire Biogéosciences, Université Bourgogne Franche-Comté, UMR CNRS 6282, Dijon, France; Broad Institute

## Abstract

We announce the draft genome sequences of two pathogenic microsporidia of European freshwater crustaceans, Thelohania contejeani (the causative agent of porcelain disease) and Cucumispora dikerogammari. Both species are implicated in mass mortalities in natural populations of their crayfish and amphipod hosts, respectively.

## ANNOUNCEMENT

Microsporidia are unicellular eukaryotes related to Fungi, specialized in intracellular parasitism (1). They are abundant in aquatic organisms, in which they can cause important disease, including crustaceans (2). Here, we sequenced the genomes of two major microsporidian pathogens of freshwater crustaceans, Thelohania contejeani (3) and Cucumispora dikerogammari (4). *T. contejeani* is the causative agent of porcelain disease in marine and freshwater decapods (5, 6). It is the most frequently encountered microsporidian parasite in European crayfish species. In the advanced phase of the disease, the abdominal muscles of infected individuals have an opaque white color. It is a chronic disease with low levels of infection in healthy populations (<2%), but it can reach up to 30% and cause population crashes (5, 6). *C. dikerogammari* is among the most recently discovered microsporidian parasites of crustaceans (4). It was originally described in the Ponto-Caspian amphipod Dikerogammarus villosus, and related parasites have since been reported in other amphipod species (7, 8). It causes severe disease in infected hosts through muscle invasion, resulting in an opaque white color (4), ultimately inducing high mortality levels (9). It is considered an emerging disease in European rivers (10).

Specimens of the crayfish Austropotamobius pallipes infected with *T. contejeani* and of the amphipod *D. villosus* infected with *C. dikerogammari* were collected in the Veude River (46.863192°N, 0.410296°E) and the Rhine River (47.814543°N, 7.546054°E), respectively. Abdominal muscles were punctured with a needle to collect microsporidian spores. Genomic DNA was directly extracted using the Qiagen DNeasy blood and tissue kit, according to the protocol for animal tissues. Paired-end libraries were constructed using the NEB Ultra II library prep kit and sequenced on an Illumina HiSeq 3000 instrument (2 × 150-bp reads) by Genome Québec, producing 232,008,634 and 252,071,596 raw paired-end reads for *T. contejeani* and *C. dikerogammari*, respectively. The raw reads were *de novo* assembled using SPAdes v3.12.0 (11) with default parameters, except for -k 21,33,55,77,99,127. The assemblies were checked for potential contamination with BlobTools v1.0 (12), MaxBin v2.2.4 (13), and PhylOligo v1.0 (14, 15). Gene structures and repeats were identified using GeneMarkS v4.30 (intronless eukaryotic parameter) (16) and RepeatModeler v1.0.8 (http://www.repeatmasker.org), respectively. Further details are available in reference 15.

**FIG 1:**
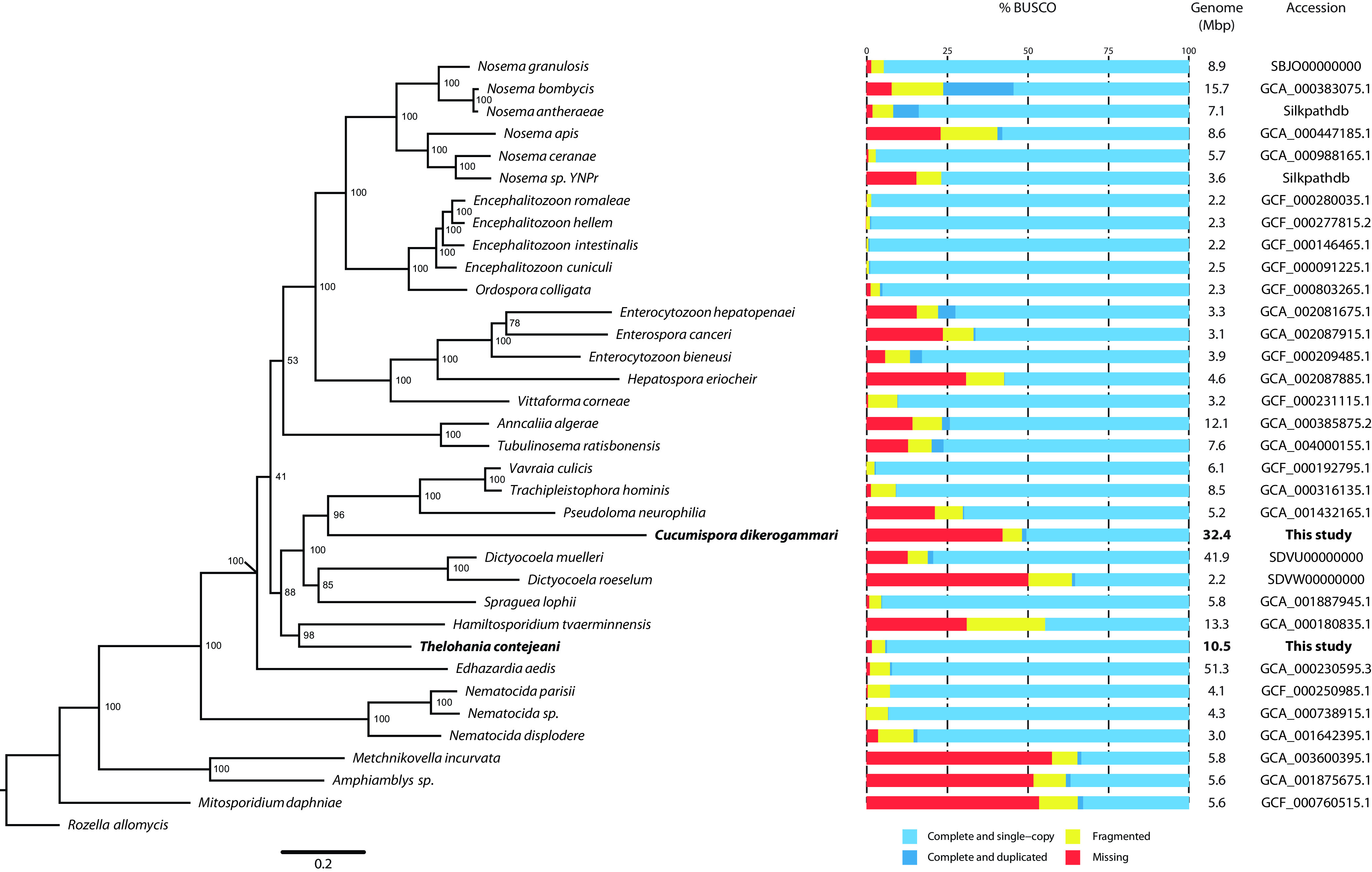
Phylogenomic analysis of microsporidia (left) along with genome assembly completeness (BUSCO, middle) and genome size and accession numbers (right). The tree was reconstructed using maximum likelihood with RAxML v8.2.9 under the LG+GAMMA model (19), based on the 68 single-copy orthologous genes (18,018 amino acids) identified in at least 31 species using OrthoFinder v2.2.7 (20). Protein sequences were aligned using MAFFT v7.299b in automatic mode (21). Individual alignments were trimmed using Gblocks (22) (-t=p; -p=n; -b3=8; -b4=2; -b5=h) and then concatenated into a single alignment using FASconCAT v1.0 (23). *Thelohania contejeani* and *Cucumispora dikerogammari* are bolded. Bootstrap values (percent) are indicated at each node (1,000 replicates). The scale bar indicates changes per site.

The *T. contejeani* assembly was composed of 1,391 contigs with an *N*_50_ value of 27,037 bp and a length of 10.4 Mbp (Table 1). Genome completeness was assessed using Benchmarking Universal Single Copy Orthologs (BUSCO) v3.0 (microsporidian set) (17), revealing that 98.3% of genes were present in the assembly. *T. contejeani* is among the most complete microsporidian genomes sequenced to date (Fig. 1). A phylogenomic analysis indicated that *T. contejeani* is related to the crustacean-borne Hamiltosporidium tvaerminnensis (18) (Fig. 1). The *C. dikerogammari* assembly was composed of 7,783 contigs with an *N*_50_ value of 9,513 bp and a length of 32.4 Mbp (Table 1). A phylogenomic analysis indicated that *C. dikerogammari* is highly divergent from the most closely related species (Fig. 1), which may help explain its BUSCO score of 57.7%.

**TABLE 1:** Assembly statistics and accession numbers of the genome sequences of *Thelohania contejeani* and *Cucumispora dikerogammari*

Metric	Data for:	
	*Thelohania contejeani*	*Cucumispora dikerogammari*
Assembly size (bp)	10,381,894	32,414,047
No. of contigs	1,391	7,783
*N*_50_ (bp)	27,037	9,513
Sequencing depth (median, bp)	3,663	1,129
G+C content (%)	26.97	26.08
Proportion of repeats (%)	32.64	36.80
No. of genes	2,865	4,599
Gene density (genes/kb)	0.28	0.14
Mean CDS^*a*^ length (bp)	1,012	875
BUSCO (*n* = 518)		
No. (%) of complete genes	488 (94.2)	268 (51.8)
No. (%) of complete and single-copy genes	485 (93.6)	261 (50.4)
No. (%) of complete and duplicated genes	3 (0.6)	7 (1.4)
No. (%) of fragmented genes	21 (4.1)	31 (6.0)
No. (%) of missing genes	9 (1.7)	219 (42.2)
GenBank accession no.	SBIQ00000000	SBJP00000000
SRA accession no.	SRR8476225, SRR8476226	SRR8495097, SRR8495098

a CDS, coding DNA sequence.

### Data availability.

The genome projects and raw sequence data sets are available at DDBJ/EMBL/GenBank and the SRA under the accession numbers provided in Table 1.
